# Picture-word interference is a Stroop effect: A theoretical analysis and new empirical findings

**DOI:** 10.3758/s13423-016-1167-6

**Published:** 2016-10-06

**Authors:** Peter A. Starreveld, Wido La Heij

**Affiliations:** 1 University of Amsterdam, Brain and Cognition, Room 0.11, P.O. Box 15915, 1001 NK Amsterdam, The Netherlands; 2 Department of Psychology and Leiden Institute for Brain and Cognition, Leiden University, Leiden, The Netherlands

**Keywords:** Stroop interference, Picture-word interference, Vincentized RT distribution

## Abstract

The picture-word interference (PWI) paradigm and the Stroop color-word interference task are often assumed to reflect the same underlying processes. On the basis of a PRP study, Dell’Acqua et al. (Psychonomic Bulletin & Review, 14: 717-722, 2007) argued that this assumption is incorrect. In this article, we first discuss the definitions of Stroop- and picture-word interference. Next, we argue that both effects consist of at least four components that correspond to four characteristics of the distractor word: (1) response-set membership, (2) task relevance, (3) semantic relatedness, and (4) lexicality. On the basis of this theoretical analysis, we conclude that the typical Stroop effect and the typical PWI effect mainly differ in the relative contributions of these four components. Finally, the results of an interference task are reported in which only the nature of the target – color or picture – was manipulated and all other distractor task characteristics were kept constant. The results showed no difference between color and picture targets with respect to all behavioral measures examined. We conclude that the assumption that the same processes underlie verbal interference in color and picture naming is warranted.

In the original Stroop task as reported by J.R. Stroop (1935), participants were required to name the ink color of a word or a nonword. The main observation in this task is that naming the color (e.g., red) of an incongruent color word (e.g., BLUE) takes more time than naming the color of a neutral stimulus (e.g., XXXXX). Since the 1960s, the Stroop task has become an important tool in cognitive psychology to study the processes underlying word production, word recognition, attention and executive control. Over the years, substantial modifications of the original paradigm were introduced. These modifications included the spatial and temporal separation of color and word (Dyer, 1971, 1973; Glaser & Glaser, 1982; Goolkasian, 1981; Hagenaar & van der Heijden, 1986; La Heij, van der Heijden, & Plooij, 2001), manipulation of the number of distractor items in a single trial (“Stroop dilution”; Kahneman & Chajczyk, 1983; Mitterer, van der Heijden, & La Heij, 2003), manipulation of the type of relation between the distractor word and the target (Klein, 1964; La Heij, Dirkx & Kramer, 1990; Neumann, 1980), the use of pictures instead of words as distractor stimuli (La Heij, Boelens, & Kuipers, 2010; La Heij & Boelens, 2011; Prevor & Diamond, 2005), and the use of pictures instead of colors as target stimuli (Glaser & Düngelhoff, 1984; La Heij, 1988; Lupker, 1979; Rosinski et al., 1975).

Many of these variants of the Stroop task resemble the original paradigm in that they typically require a verbal reaction to a (often, but not always, nonverbal) target stimulus in the context of an (often, but not always, verbal) distractor. That is probably the reason why these tasks are typically referred to as “Stroop-like paradigms.” Of course, this designation does not imply that the processes underlying the interference effects observed in the various tasks are necessarily identical. In fact, it has been shown that at least some of the observed interference effects have different causes. For example, La Heij et al. (2010) and La Heij and Boelens (2011) presented evidence that color-object interference in children 5–7 years of age (naming the color of an object takes them longer than naming the color of an abstract form), is most probably not due to interference at a level of response selection, but to an earlier problem in selecting the correct task when two nameable stimuli (color and picture) are presented simultaneously on a screen. In line with the authors’ interpretation of color-object interference in terms of immature executive control, the interference effect (in contrast to Stroop color-word interference) was shown to be virtually absent in adult participants (La Heij & Boelens, 2011).

However, with respect to two variants of the Stroop task, the original color-naming task and the picture-word interference task (henceforth the PWI task; e.g., Glaser & Düngelhoff, 1984; Lupker, 1979; Rosinski et al., 1975), many researchers agreed that they are very similar with respect to the mechanisms involved (see, e.g., McLeod, 1991; Van Maanen, Van Rijn, & Borst, 2009). With respect to the PWI task, Glaser and Düngelhoff (1984), for instance, argued: “The color of the Stroop stimulus may be considered the limiting case of the picture component.” (p. 640). This idea is supported by the many similarities in empirical findings obtained with the two paradigms and the – often implicit – assumption that processes underlying the naming of a color and a picture do not differ in a crucial way. Especially in research on language production the results obtained with two tasks are used interchangeably (e.g., Mulatti & Coltheart, 2014; Roelofs, 2003; Roelofs & Piai, 2013).

However, Dell’Acqua, Job, Peressotti, and Pascali (2007) took issue with the idea that Stroop interference and PWI result from identical underlying processes. They reported the results of a psychological refractory period (PRP) study in which the PWI task was combined with a second, manual response task in order to determine the locus of the interference effects observed. Because the pattern of results obtained differed from the one observed with Stroop (color-word) interference in a study by Fagot and Pashler (1992), Dell’Acqua et al. concluded that PWI is not a Stroop effect. Whereas Stroop interference is probably localized at a response selection stage, they argued, PWI might be localized at an earlier processing level. This conclusion has been criticized on the basis of substantial methodological differences between the authors’ PWI experiment and the Fagot and Pashler (1992) color-naming task. Moreover, in two studies, the findings obtained by Dell’Acqua et al. (2007) were not replicated (Piai, Roelofs, & Schriefers, 2014; Schnur & Martin, 2012).

The present paper addresses the issue of whether the PWI task is a Stroop-like task, but uses a somewhat different approach. We first examine the definitions of Stroop interference and picture-word interference as used in the literature. Next, the interference effects observed in both paradigms are discussed in terms of various *components of interference*, induced by various distractor characteristics. On the basis of this analysis, we conclude that the “overall Stroop interference effect” and the “overall PWI effect” contain identical interference components and only differ in the relative contribution of each of these components.

Finally, we report an experiment that was designed to examine an important difference between the two paradigms that – with the exception of the Van Maanen et al. (2009) study – did not receive much attention in the literature: the difference between naming a color (in the Stroop task) and naming a picture (in the PWI task). To reliably compare color and picture naming, all other factors in which the original paradigms differed were kept constant: the number of semantic domains involved, the size of the target set and the presence or absence of distractor words in the response set. To anticipate our findings: with respect to all behavioral data examined (e.g., the size of the interference effect, the time-course of the interference effect across stimulus-onset asynchrony (SOA) intervals, the reaction time (RT) distributions of interference effect, the standard deviations of the RTs in the various conditions, and the effect of practice), color naming and picture naming produced virtually identical results.

## Stroop versus picture-word interference (PWI): The definitions

A first problem in comparing the Stroop effect and the PWI effect is that there are no universally accepted definitions of the two effects. The term “Stroop effect” is often used to denote the difference between an incongruent condition (e.g., “RED” in the color blue) and a neutral condition in which participants name the color of a series of characters (e.g., “XXXXX” in the color blue) or color patches. However, it is not hard to find studies in which, instead, the Stroop effect is defined as the difference between an incongruent condition and a congruent condition (e.g., “RED” in the color red). In fact, Fagot and Pashler (1992) used this definition in the PRP experiment that Dell’Acqua et al. (2007) referred to in their comparison of Stroop interference and PWI. The term “PWI effect” is equally ill-defined: it often refers to the difference between picture-naming latencies in the context of a semantically related word and a neutral distractor (e.g., a series of Xs), but in some studies, including the Dell’Acqua et al. study, the PWI effect is defined as the difference in picture-naming latencies between a semantically related and unrelated distractor word condition. In this paper we will use the definition that is most common: the difference between an incongruent condition, in which target and distractor are semantically related (e.g., “RED” in blue or the word “BIKE” superimposed on the picture of a car) and a neutral condition, in which the distractor is a nonword (e.g., a series of Xs).

## Components of interference

Related to the issue of definitions is the important issue of the components the overall interference effect can be broken down into. Previous studies have repeatedly shown that Stroop interference and PWI can be attributed to a number of different characteristics of the distractor word. La Heij (1988; see also La Heij, van der Heijden, & Schreuder, 1985) discussed four independent components and determined their contribution to the overall interference effect in a PWI task. To that end, six target pictures were selected from two semantic categories (e.g., the categories “fruit” and “body parts”, with the target pictures of a pear, cherry, banana, finger, mouth, and nose). Six distractor conditions were created: the target picture of (for example) a pear was accompanied by the distractor word CHERRY (semantically related to the target pear, member of one of the two relevant semantic categories – fruit and body parts – and one of the six response words; condition REL/RS), NOSE (semantically unrelated to the target pear, member of one of the two relevant categories – fruit and body parts – and one of the six response words; condition UNR/RS), LEMON (semantically related to the target pear, member of one of the two relevant categories – fruit and body parts – and not one of the six response words; condition REL/NRS), HAND (semantically unrelated to the target pear, member of one of the two relevant categories – fruit and body parts – and not one of the six response words; condition UNR/NRS), DOOR (semantically unrelated to the target pear, not a member of one of the two relevant categories – fruit and body parts – and not one of the six response words; condition IRR), or a string of Xs (a nonword; condition CONTR).

Table 1 shows the relative contributions of four interference components (the two experiments in La Heij, 1988, combined) to the overall interference effect (defined as REL/RS – CONTR) : (a) the distractor word is (or is not) an eligible response in the experiment (“response-set membership”; defined as [REL/RS + UNR/RS]/2 – [REL/NRS + UNR/NRS]/2), (b) the distractor word is semantically related or unrelated to the accompanying target (“semantic relatedness”; defined as [REL/RS + REL/NRS]/2 – [UNR/RS + UNR/NRS]/2), (c) the distractor word is (or is not) a member of the two semantic categories from which the targets are selected (“task relevance”; defined as UNR/NRS - IRR) and (d) the distractor is a meaningful word or is a meaningless and unpronounceable letter string, like “XXXXX”(“lexicality”; defined as IRR - CONTR). We discuss these components in turn.

**Table 1:** Four components of interference and their estimated relative contributions to the overall interference effect in a picture-word interference (PWI) task with six target pictures selected from two semantic categories reported by La Heij (1988)

Interference component	Relative contribution
Distractor is an eligible response (“response-set membership”)	20 %
Distractor is semantically relevant in the task (“task relevance”)	29 %
Distractor is semantically related to the target (“semantic relatedness”)	14 %
Distractor is a pronounceable and meaningful letter string (“lexicality”)	37 %
Sum of the four components	100 %

*Note*. See text for details of the calculations

A first component of Stroop interference is due to the distractor word being an eligible response in the experiment or not (“response-set membership”). In a typical Stroop task, in which the target colors red, blue, green, and yellow are used, a distractor word like “red” is not only task-relevant and semantically related to the accompanying target color (e.g., blue), but is also the name of one of the other targets and – for that reason – is repeatedly produced as a response in the experiment. In a number of Stroop studies it has been shown that color words that are eligible responses induce more interference than color words that are not (e.g., a distractor word like “brown”; Fox et al. 1971; Klein, 1964; Lamers, Roelofs, & Rabeling-Keus, 2010; Proctor, 1978). Not surprisingly, it has also been shown that the size of this response-set effect decreases when the target-set size increases. In a simple picture-naming task, La Heij and Vermeij (1987) examined target-set sizes of two, four, and eight stimuli (responses) and reported response-set membership effects of 17 ms, 9 ms and −3 ms, respectively. The implication of these findings for our current discussion is that the typical Stroop interference effect (with four target colors) contains a substantial response-set membership effect that is probably very small or absent in the typical PWI task (with 20 target pictures or more). This is because in the PWI task the distractor words are not eligible responses (they do not denote one of the target pictures) or – if they are – the set of targets (responses) is too large to result in a measurable effect. In the atypical PWI experiments reported by La Heij (1988), in which only six target pictures were used, the relative contribution of this component to the overall interference effect was 20 % (see Table 1). In the study by Proctor (1978), in which four target colors were used, this response set effect accounted for 27 % of the overall Stroop interference effect.

A second, little investigated, component of Stroop interference can be attributed to the “semantic relevance” of the distractor word in the task at hand. In a Stroop color-naming task with red, green, and blue as the target colors, distractor words like “yellow” and “brown” – although not members of the response set – are semantically very relevant, whereas distractor words like “horse” and “coat” are not. Neumann (1986) coined this effect “task relevance.” In the orthodox Stroop task, in which all stimuli are selected from one semantic category (color), the factor task relevance is completely confounded with the factor semantic similarity between target and distractor. To determine whether task relevance has a measurable effect, Neumann (1986) combined a color-naming and dot-counting variant of the Stroop task. Likewise, La Heij (1988) used a PWI task in which three target pictures were selected from each of two semantic categories (e.g., fruit and body parts). This allowed for the use of distractor words that were (a) task relevant and semantically related (e.g., the picture of a pear with the word “lemon” superimposed), (b) task relevant but semantically unrelated (e.g., the picture of a pear with the word “hand” superimposed), and (c) neither task relevant nor semantically related (e.g., the picture of a pear with the word “door” superimposed). As shown in Table 1, task relevance turned out to be responsible for a substantial part (29 %) of the overall PWI effect. Analogous to the effect of response-set membership, the effect of task relevance most probably decreases when the number of semantic domains from which the targets are selected increases. In the Stroop task (only one relevant category) it will be maximal, whereas in a typical PWI task with many pictures taken from many semantic categories, the effect will be small or absent. 1

The third component of Stroop interference to be discussed here is the effect of a semantic relation between the target and the accompanying word in a single trial. Again, the size of this effect cannot be estimated in the traditional Stroop task, because the factors semantic relatedness and task relevance are confounded. As discussed above, La Heij (1988) disentangled the two effects in a PWI task and found that semantic relatedness accounted for 14 % of the overall interference effect (see Table 1). In the original Stroop task it is only possible to determine the combined effect of semantic relatedness and task relevance by comparing, for example, a distractor word like “brown” (semantically related, task relevant, but not part of the response set) with a distractor word like “bottle” (semantically unrelated and task irrelevant). In the study by Proctor (1978), this combination of semantic effects was responsible for 46 % of the overall Stroop interference effect. In La Heij’s (1988) PWI tasks this percentage was 43 %.

The final component of Stroop interference in Table 1 is the effect of lexicality, caused by a distractor word that is not semantically related to the target, not relevant in the task at hand, and not part of the (small) response set. That is, it is the effect of a distractor word like “bottle” in comparison with a neutral distractor (e.g., a series of Xs) on color naming. In the Stroop task reported by Proctor (1978), this interference effect amounted to 27 % of the overall interference effect. In La Heij (1988) the corresponding percentage was 37 %.

This analysis makes it very likely that the Stroop interference effect and the picture-word interference effect consist of the same set of interference components but differ in the relative contributions of each of these components. The size of these components is dependent on the experimenter’s choices with respect to (a) the number of semantic domains from which targets are selected, (b) the number of different target stimuli, and (c) the use of target names as distractor words or not. Viewed in this way, the traditional Stroop task and the traditional PWI task are simply two extremes on a continuum of interference tasks.

## Dell’Acqua et al. (2007) revisited

Given this theoretical analysis, let us take another look at the study of Dell’Acqua et al. (2007). These authors set out to compare PWI and Stroop interference using a PRP paradigm. What they actually compared, however, is the component “semantic interference” obtained in a PWI experiment that they performed (a component that was responsible for 14 % over the overall PWI effect in the study of La Heij, 1988; see Table 1) with a Stroop interference effect reported by Fagot and Pashler (1992), consisting of all four components in Table 1 augmented by a fifth component, a facilitation effect induced by a congruent distractor word (e.g., RED in red). It will be clear that the results of such a comparison will be hard to interpret (cf. Piai et al., 2014).

To compare Stroop interference and PWI, one should first decide what particular aspect (interference component or components) one is interested in and match the two paradigms with respect to all other characteristics. In their discussion, Dell’Acqua et al. (2007) suggested that a crucial difference between the two tasks may be in the use of colors and color names in the Stroop task versus pictures and picture names in the PWI task. They suggested that “semantic activation mediated by color words and semantic activation mediated by real-world concepts differ in terms of temporal persistence” (p. 722). We agree that if the interference effects in the two paradigms behave differently, the nature of the target – color versus picture – is a plausible cause. For example, whereas pictures and picture names are assumed to activate a rich network of conceptual representations, colors – being visual attributes of objects – may induce a qualitatively different or more restricted activation in the conceptual system. However, whether such differences exist when the contribution of each component discussed above is similar for each task remains an empirical question.

## The present study

In the experiment to be reported we compared interference in color and picture naming using two tasks that were matched with respect to all components discussed above. In the Stroop task four colors were used as targets and the names of these colors were used as incongruent distractor words. In the PWI task four pictures were used that were selected from a single semantic category (mammals) and the names of these pictures were used as incongruent distractor words. In the neutral or baseline condition, the targets we presented were in combination with a series of Xs. One other difference between traditional Stroop and PWI was also eliminated: the spatial integration of color and distractor in the Stroop task (by presenting a colored word) and the spatial separation of target and distractor in the PWI task (by superimposing word and picture). To that end, in the Stroop task the color was presented as colored lines (see Method) covering approximately the same spatial area as the target picture in the PWI task.

Instead of just looking at the Stroop and PWI effect at a single SOA interval (as Dell’Acqua et al., 2007, and Fagot & Pashler, 1992, did), we set out to obtain a complete picture of the development of the interference effect. To that end, we manipulated the SOA interval between the presentation of the two components of each stimulus. For each task we used five SOA intervals: −200 ms, −100 ms, 0 ms, 100 ms, and 200 ms (see, e.g., Glaser & Glaser, 1982, for a similar manipulation). We analyzed the time course of the size of the interference effect across these SOA intervals, and even across the RT distributions (using Vincentized cumulative distribution curves) at each of these SOA intervals to look for differences between the tasks.

The sizes of the RTs are not the only interesting characteristics of the data obtained in color-naming and picture-naming tasks. Stroop (1935) already noted that the incongruent condition showed a much larger standard deviation (*SD*; 18.8 s) than the neutral condition (10.9 s; note that Stroop used solid colored squares without a distractor in the neutral condition and measured total response time for a series of stimuli). Therefore, we also analyzed the time course of the *SD*s of the conditions across the SOA intervals to look for differences between the tasks.

Finally, Stroop (1935) reported an effect of practice on the size of the interference effect, showing a decrease of the effect for eight successive days of practice. Because we presented five consecutive series (each exploring a different SOA) to each participant, we also manipulated the amount of practice. Hence we were able to examine the effect of this manipulation on the size of the interference effects and to examine possible differences between the tasks with respect to practice.

## Method

### Participants

A total of 43 volunteers participated in the experiment; data for one participant were lost due to an error made by the experimenter. Most participants were University of Amsterdam students and their mean age was 20.3 years (*SD* = 2.46). More than half of the participants (N=26) received course credit, the remaining participants were unpaid volunteers. None of the participants was color-blind and they all had normal or corrected-to-normal vision. Half of the participants were randomly assigned to the Stroop color-naming task, the others participated in the PWI task.

### Materials

Both tasks contained four targets selected from a single semantic category: colors in the Stroop task and mammals in the PWI task. To prevent differences in the moment of voice-key triggering, the names of the targets in the two tasks were matched with respect to their first phoneme. The four target colors selected were *groen* (green), *paars* (purple), *rood* (red), and *bruin* (brown). The corresponding names of the four target pictures were *geit* (goat), *paard* (horse), *rat* (rat), and *beer* (bear). The pictures were black line drawings presented on a white background. To increase the similarity between both tasks, the targets in the Stroop task consisted of the (colored) pictures used in the PWI task, scrambled into an unrecognizable set of line fragments. The distractor words in the incongruent condition were the names of the target colors and pictures. The distractors in the neutral (baseline) condition of both tasks were a series of five Xs (XXXXX). All distractors were displayed in gray letters (Arial font, size 28; the font color was defined by a value of 100 for all three RGB channels on a scale of 0–255) at the point of fixation, in the center of the target stimuli. Five SOA conditions were used: the distractor was presented before the target (at SOA interval= −200 ms and SOA interval= –100 ms), simultaneously with the target (SOA interval= 0 ms) or after the target (SOA interval= 100 ms and SOA interval= 200 ms).

### Apparatus

Stimulus presentation and recording of the responses was controlled by Presentation® software (www.neurobs.com) running on a fast Windows 7 PC. Stimuli were presented on a BenQ 3d monitor with a refresh frequency of 100 Hz and voice onset was recorded to the nearest ms using a microphone.

### Procedure

Participants were run individually in a dimly illuminated room. They were seated in front of the monitor at reading distance. After reading the information form and instructions and signing the informed consent form, the participant received four practice series in which each target stimulus was presented three times, resulting in a total of 12 trials per practice series. In the first practice series, the experimental targets were presented in combination with the correct target name. In the second practice series, the experimental targets were presented in isolation. In the third practice series, they were presented in combination with a series of five Xs, and in the final practice series they were presented in combination with the unrelated “practice” distractors *kast* (cupboard), *jas* (coat), *oog* (eye), and *vork* (fork). By the end of the practice series, participants were familiar with both the targets and the task.

Next, the participants received the experimental series. SOA interval conditions were blocked and each participant received a different order of SOA interval blocks (according to Latin squares based on a Williams design, Williams, 1949). Each block consisted of five series of 24 trials (resulting from the four targets, each combined with three possible incongruent distractor words and each combined three times with the string XXXXX). Each trial involved the following sequence: a black fixation point appeared in the center of the screen for 500 ms. Next the stimulus combination (distractor first in the negative SOA interval conditions, target and distractor simultaneously in SOA interval condition of 0 ms, target first in the positive SOA interval conditions) appeared and remained on the screen until a response was registered or 2,000 ms had elapsed after target presentation. After each response, the experimenter determined which of the following conditions applied: (a) correct response and correct triggering of the voice key, (b) correct response, but incorrect triggering of the voice key (e.g., due to non-speech sounds (e.g., mouth clicks) or low speech volume), or (c) incorrect response (including hesitation sounds). The experimenter then entered a corresponding code for each situation.

## Results

The following data trimming procedure was used: RTs of incorrect responses (color naming 2.4 %; picture naming 2.3 %), RTs of trials in which the voice key was not triggered appropriately (color naming 2.5 %; picture naming 2.5 %), RTs smaller than 300 ms (probably reflecting anticipations or mouth clicks; color naming 0.1 %; picture naming 0.3 %), and RTs larger than 1,500 ms (color naming 0.13 %; picture naming 0.17 %) were removed. The remaining RTs were used in the analyses of the RTs. Table 2 shows the mean RTs for the two experimental conditions in the color-naming and picture-naming task in each of the SOA interval conditions.

**Table 2:** Results per stimulus onset asynchrony (SOA) interval for the color-naming and picture-naming task

Task	Condition	SOA interval									
		−200		−100		0		100		200	
		RT	e%	RT	e%	RT	e%	RT	e%	RT	e%
Color naming	INC	619	2.1	620	3.3	639	4.7	637	4.4	597	2.9
	NEU	584	1.3	580	1.3	579	1.4	587	1.0	581	0.7
	INT	35	0.8	40	2.0	61	3.3	51	3.4	15	2.2
Picture naming	INC	629	2.1	644	2.2	663	4.4	663	5.3	603	3.2
	NEU	588	1.2	585	1.9	591	1.1	601	1.0	590	1.5
	INT	41	1.0	59	0.3	72	3.3	62	4.3	12	1.7

*Note. SOA* stimulus onset asynchrony, *INC* incongruent, *NEU* neutral, *INT* INC – NEU, *RT* mean reaction time, *e*% error percentage

We first analyzed the RTs per SOA interval per task, the typical procedure in Stroop- and PWI research (e.g., Glaser & Düngelhoff, 1984; Glaser & Glaser, 1982; Starreveld & La Heij, 1996). Second, we report a detailed analysis of the RT distributions involved. Third, we analyzed the *SD*s per SOA interval and per task. Fourth, to evaluate the effect of practice for both tasks, we analyzed the RTs per series. Finally, we report on the errors made in each task. In all analyses of variance (ANOVAs) reported below, Greenhouse-Geisser corrections were performed when Mauchly’s test of sphericity proved significant.

### Overall reaction-time (RT) analyses

**Fig. 1:**
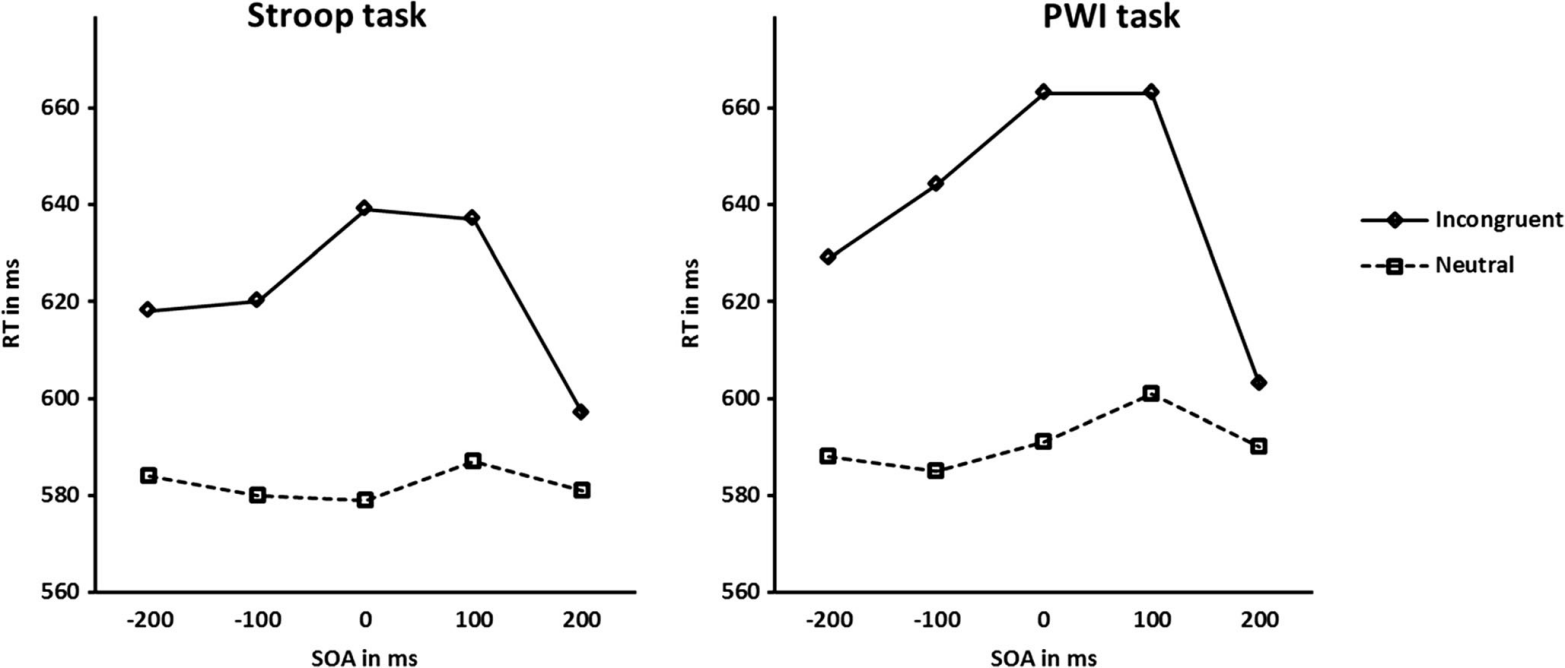
Mean reaction times (RTs) at all stimulus onset asynchrony (SOA) intervals for the incongruent and neutral conditions of the Stroop task and the picture-word interference (PWI) task

For ease of interpretation, Fig. 1 displays the mean RTs obtained for the color-naming and picture-naming tasks separately. A repeated measures ANOVA was performed on the mean RTs with context condition (incongruent vs. neutral) and SOA interval (−200 ms, −100 ms, 0 ms, 100 ms, and 200 ms) as within-participant variables and task (color-naming vs. picture-naming) as between-participants variable. This analysis showed a significant main effect of context condition, *F*(1, 40) = 125.6, *p* < .001, *MSE* = 1676.2. As expected, participants were slower in the incongruent conditions (*M* = 631 ms) than in the neutral conditions (*M* = 587 ms). The analysis also showed a significant main effect of SOA, *F*(2.7, 160) = 4.98, *p* < .005, *MSE* = 2272.3, reflecting different mean RTs at different SOAs. Finally, the interaction between condition and SOA interval was significant, *F*(4, 160) = 20.4, *p* < .001, *MSE* = 417.9. Inspection of Fig. 1 shows that the amount of interference obtained varies with SOA interval (the interference effect amounted to 38, 49, 66, 56, and 14 ms for the SOA interval conditions of −200, −100, 0, 100, and 200 ms, respectively), and reached a maximum at SOA interval 0 ms. The SOAs used in the experiments therefore seem to encompass most of the interval in which interference in these tasks can be obtained.

Importantly, the effect of the variable task was not significant, nor were any of the first- and second-order interactions involving task (all *p*s > .24). The latter results indicate that both the RTs and the interference effects obtained in the Stroop color-naming task and the PWI task were similar in size and showed a highly similar development across SOA intervals.

### RT distribution analyses per stimulus-onset asynchrony (SOA) per task

To examine possible differences in RT distributions obtained with the two tasks, the rank-ordered correct naming latencies of all participants per context condition (incongruent and neutral) and SOA interval were divided into quintiles (i.e., five bins, each containing one-fifth of the RTs). Next, mean RTs were computed for each quintile. By averaging these means across participants in both tasks, Vincentized cumulative distribution curves were obtained (Ratcliff, 1979; see e.g., Roelofs, 2008, for an application in language production research). Figure 3 shows the obtained distributions of each of the SOA interval conditions, context condition and tasks.

**Fig. 2:**
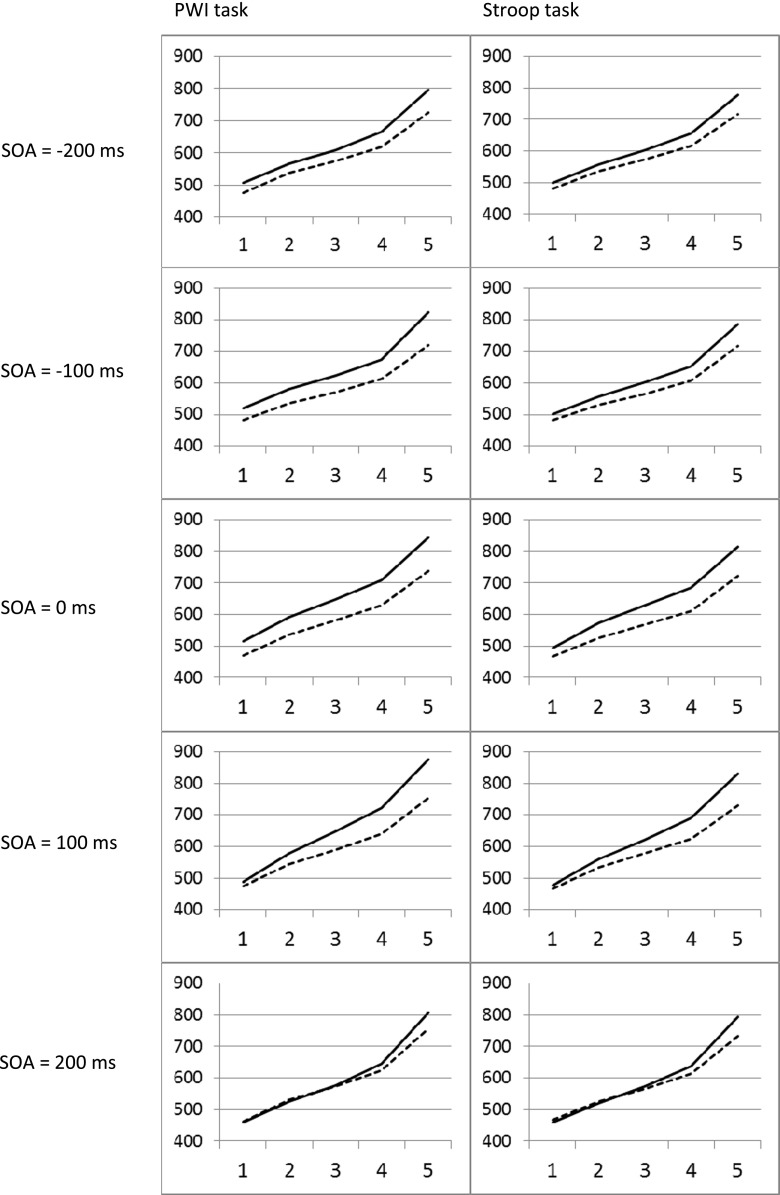
Mean reaction time (RT) distributions at all stimulus onset asynchronies (SOAs) for the incongruent and neutral conditions of the picture-word interference (PWI) task and the Stroop task. The x-axes represent the five RT bins used, the y-axes represent RTs in ms. Solid lines represent RTs from the incongruent condition, dotted lines represent RTs from the neutral condition

A repeated measures ANOVA performed on these RTs with SOA interval (−200 ms, −100 ms, 0 ms, 100 ms, and 200 ms), context condition, and quintiles (1–5) as within-participants variables and task (color- vs. picture-naming) as a between-participants variable showed a main effect of SOA, *F*(2.7, 160) = 4.96, *p* < .005, *MS*_*e*_ = 11367.7, context condition, *F*(1, 40) = 126.04, *p* < .001, *MS*_*e*_ = 8296.5, and quintile, *F*(1.1, 160) = 665.29, *p* < .001, *MS*_*e*_ = 7625.7. Significant first-order interactions were obtained for SOA interval × context condition, *F*(4, 160) = 19.426, *p* < .001, *MS*_*e*_ = 2066.5, SOA interval × quintiles, *F*(4.6, 640) = 13.56, *p* < .001, *MS*_*e*_ = 707.4, and context condition × quintiles, *F*(1.4, 160) = 64.06, *p* < .001, *MS*_*e*_ = 1057.3. Finally, also the second-order interaction between SOA, context condition, and quintiles reached significance, *F*(6.1, 640) = 3.99, *p* < .005, *MS*_*e*_ = 405.9. Inspection of Fig. 2 shows that this latter interaction is caused by a tendency for larger interference effects at the last bins of the RT distributions, a tendency that grows larger for increasing SOAs.

All effects reported above show that our tasks were not only sensitive enough to pick up interference effects in the overall RT distributions, but even sensitive enough to pick up differences of these effects for the various quintiles of these distributions. In that light, the most important result of the present analysis was that, despite the fact that our RT measure showed large sensitivity to the complex development of the context effect over SOAs and quintiles, none of the interactions involving the factor task (color-naming vs. picture-naming) reached significance (all *p* > .23).

### Standard deviation analyses per SOA per task

**Fig. 3:**
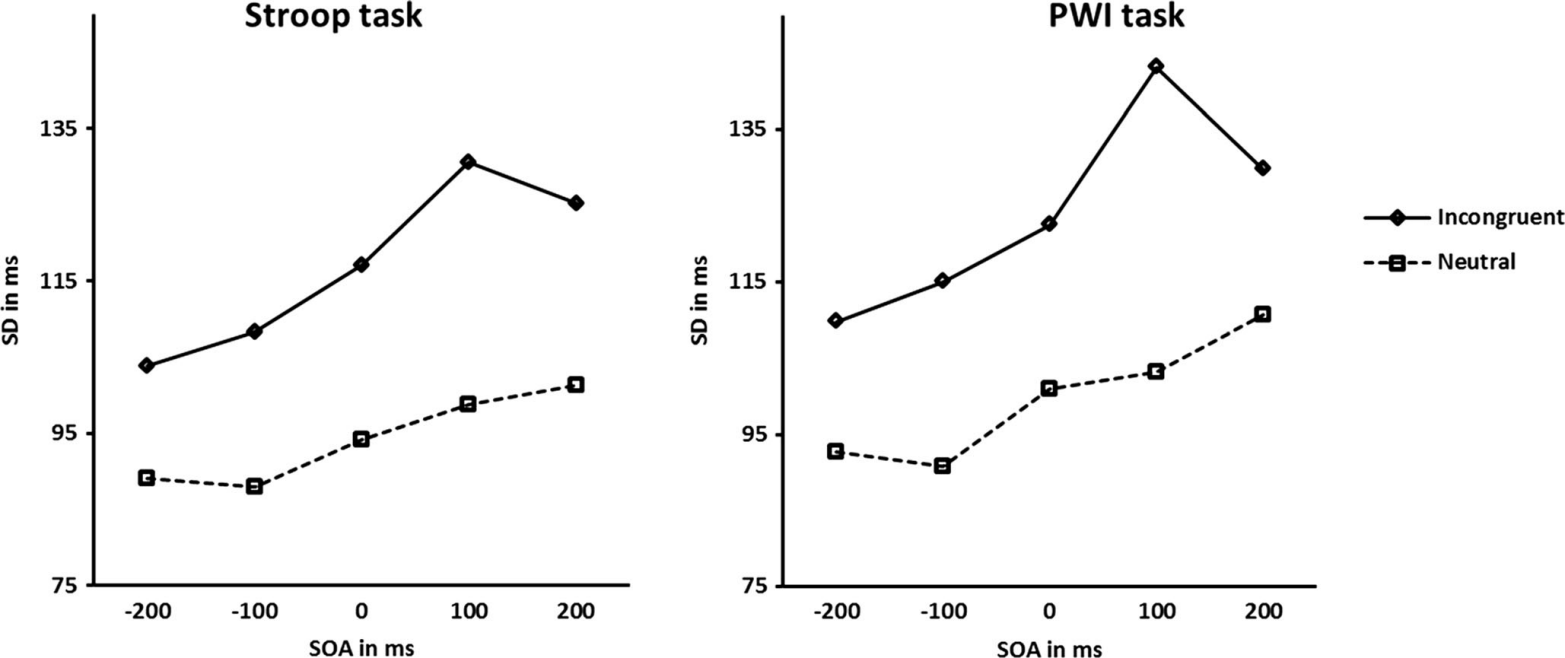
Mean standard deviations (*SD*s) at all stimulus onset asynchronies (SOAs) for the incongruent and neutral conditions of the Stroop task and the picture-word interference (PWI) task

As noted in the introduction, Stroop (1935) already noted that RTs from the neutral condition showed a much smaller *SD* than the RTs from the incongruent condition. The next analysis was run to evaluate whether we obtained a similar result and whether the sizes of the *SD*s involved differed for the two tasks, color naming and picture naming. To that end, we calculated the *SD*s for each participant for each task and each condition at each SOA. For ease of interpretation, Fig. 3 displays the mean *SD*s involved for each task separately.

A repeated measures ANOVA was performed on the *SD*s with condition (incongruent vs. neutral) and SOA interval (−200 ms, −100 ms, 0 ms, 100 ms, and 200 ms) as within-participant variables and task (color-naming and picture-naming) as between-participants variable. The analysis showed a significant main effect of condition, *F*(1, 40) = 77.8, *p* < .001, *MSE* = 746.5. The mean *SD* in the incongruent conditions (*M* = 120 ms) was larger than in the neutral conditions (*M* = 97 ms). The analysis also showed a significant main effect of SOA, *F*(3.3, 160) = 13.4, *p* < .001, *MSE* = 83198.5, reflecting different mean *SD*s at different SOAs. Finally, the interaction between condition and SOA interval was significant, *F*(4, 160) = 4.0, *p* < .005, *MSE* = 283.9. Inspection of Fig. 3 shows that the *SD*s increase with increasing SOA interval values, but decrease again at SOA interval 200 ms.

Importantly, also in the analysis of the *SD*s, the effect of the variable task was not significant, nor were any of the first- and second-order interactions involving task (all *p*s > .44). These results indicate that both the *SD*s and the difference in *SD*s between the incongruent and neutral conditions obtained in the Stroop color-naming task and the PWI task were similar in size and showed the same development across SOA intervals.

### RT analyses per series per task

**Fig. 4:**
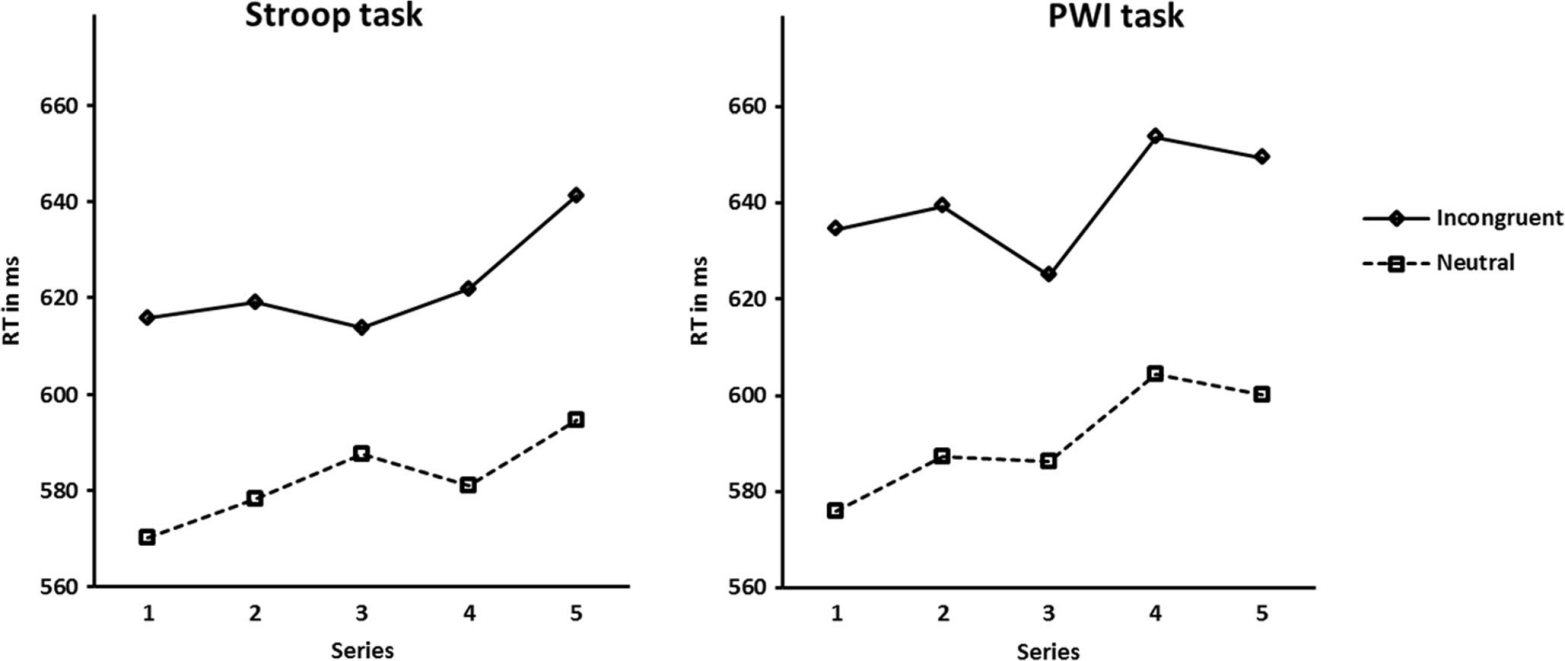
Mean reaction times (RTs) at all series for the incongruent and neutral conditions of the Stroop task and the picture-word interference (PWI) task

Participants performed the task presented to them in five series, each testing a particular SOA. To evaluate the effect of practice on RTs, we calculated the mean RTs collapsed over SOAs at each series. Because the order of SOA interval presentation was balanced across participants, collapsing the data over SOAs results in comparable data sets for each series. These data sets might reveal a general increase or decrease in response speed to the targets throughout the experiment as a result of practice. Figure 4 displays the mean RTs involved for each task separately.

A repeated measures ANOVA was performed on the mean RTs with condition (incongruent vs. neutral) and series (first, second, third, fourth, and fifth) as within-participant variables and task (color naming and picture naming) as between-participants variable. This analysis showed a significant main effect of condition, *F*(1,40) = 125.7, *p* < .001, *MSE* = 1672.7. This overall effect is identical, of course, to the effect obtained in the corresponding analysis per SOA. In addition, the analysis showed a significant main effect of series, *F*(2.8, 160) = 2.98, *p* = .038, *MSE* = 2355.8, reflecting different mean RTs at different series.Inspection of Fig. 4 shows that RTs increased slightly with increasing practice (*M* = 599, 606, 603, 615, 621, for series 1, 2, 3, 4, and 5, respectively), probably indicating an effect of fatigue on behalf of the participants. Finally, the interaction between condition and series was not significant, *p* = .13.

Importantly, the effect of the variable task was not significant, nor were any of the first- and second-order interactions involving task (all *p*s > .24). The latter results indicate that both the RTs and the interference effects obtained in the Stroop color-naming task and the PWI task were similar in size and showed the same development as a result of practice.

### Error analysis

The error percentages were considered too small to allow for a meaningful analysis. The figures shown in Table 2 show – numerically – the usual pattern: more errors in the incongruent condition than in the neutral condition, especially in the SOA interval conditions 0 ms and 100 ms.

## Discussion

The data for both the Stroop task and the picture-word interference task showed clear interference effects (incongruent vs. neutral), both in RTs and in *SD*s. These effects varied in size with SOA. The RT measurements also showed differences between the various quintiles of the RT distribution at each SOA. These results clearly reveal the sensitivity of the RT measure. However, no significant differences between the two tasks emerged for all dependent and independent variables examined: mean overall RT, mean RT per condition, mean RT per SOA, mean RT per condition per SOA, mean *SD* per condition, mean *SD* per SOA, mean *SD* per condition per SOA, and mean RT per condition at every quintile of each SOA interval used. In addition, although we also found clear effects of the amount of practice on mean overall RTs and mean RTs per condition (again indicating that our RT measure was sensitive to the cognitive processes involved in performing the tasks), once more no significant differences between the tasks emerged on mean RTs, mean RTs per condition, and mean RTs per condition per series.

We cannot but conclude that performance in the two tasks was virtually identical. On the basis of these findings there is no reason whatsoever to assume that Stroop- and picture-word interference differ in their underlying processes, as might possibly have arisen due to a difference in the nature of the target: color versus picture.

Our conclusion, based on behavioral data, seems in line with findings obtained by cognitive neuroscientists. Piai, Roelofs, Acheson, and Takashima (2013), for instance, showed that incongruent trials in both the PWI task and the Stroop task were associated with activation of a portion of the dorsal anterior cingulate cortex (ACC) and with activity in the anterior-superior temporal gyrus (STG). Moreover, in a MEG study in which participants performed a PWI task, Piai, Roelofs, Jensen, Schoffelen, and Bonnefond (2014) observed the same midline frontal theta signature that was earlier found by Hanslmayr et al. (2008) in a study in which participants performed the Stroop task. Such findings suggest common mechanisms involved in the two tasks.

Given the assumption that common mechanisms are involved in Stroop and PWI tasks, a next question concerns the locus of the underlying mechanism that causes the effect of incongruent distractors in both tasks. Most researchers (including Dell’Aqua et al., 2007) agree that the Stroop interference effect is localized at a relatively “late” (postperceptual) locus. So, on the basis of our findings, the conclusion seems warranted that PWI also has a “late” locus. Our present data do not adjudicate, however, between a locus at the level of lexical selection (e.g., Roelofs, 2003; Starreveld & La Heij, 1995, 1996) or a locus at an output (monitoring) level (e.g., Mahon, Costa, Peterson, Vargas, & Caramazza, 2007, but see Starreveld, La Heij, & Verdonschot, 2011, who argued against an output level interpretation of semantic interference in the PWI task).

In our present study, we did not find differences in the size of the interference effects induced by incongruent distractors in the Stroop task and the PWI task despite the fact that the four targets in the Stroop task were taken from the rather small set of frequently used color words, whereas the four targets in the PWI task were taken from the much larger set of mammals. In our view, a likely explanation of this finding is that it is not the number of category exemplars that is important, but the number of target stimuli (responses) used in a particular task. Such an interpretation suggests that it is the capacity of short-term memory that is crucial. Some support for this idea is La Heij and Vermeij’s (1987) finding that the effect of response-set membership decreased with increasing set size and was absent with a set size of eight.

One unexpected result of the present study was that RTs increased, albeit slightly, with practice, both for the color-naming task and for the picture-naming task. In his seminal paper, Stroop (1935) reported a decrease of RTs over several days of practice with the incongruent color-naming task, a result that has been replicated since with several variants of the Stroop task (e.g., Flowers & Stoup, 1977; Harbeson, Krause, Kennedy, & Bittner Jr., 1982; MacLeod, 1998). The most important difference between these studies and our present study is that we examined the effect of practice during one session, whereas the other studies examined the effect of practice in sessions spread out over successive days. Therefore, we suggest that the fact that we found a slight increase in naming times can be attributed to this difference. It seems reasonable to assume that our participants experienced an increase of fatigue during the one session, whereas participants who are allowed a break of 1 day between sessions do not experience such an increase in fatigue. One study (Dulaney & Rogers, 1994) in which a decrease of RTs as a result of practice was reported did use only one practice session, but (a) the practice consisted of naming incongruent stimuli only and (b) during the experiment several stimuli were presented at once, so that participants needed to “orient to the first stimulus on the screen and develop a scanning pattern going from left to right, line by line” (Dulaney & Rogers, 1994, p. 478). In contrast, we presented both incongruent and neutral stimuli during the whole experiment and we presented our stimuli one at a time. Such procedural differences might well be responsible for the differences in the practice effects obtained. In sum, although we found a different effect of practice than has often been reported in the literature, we think that this difference can be attributed to the specifics of our procedure. What remains is that our practice effect was statistically identical for both the color-naming task and the picture-naming task.

As discussed in the introduction, differences in the size of specific interference components in traditional realizations of the Stroop task and the picture-word interference task should most probably be linked to differences in experimental set-up. In the traditional Stroop task, with only few targets selected from a single semantic category, an important part of the overall interference effect is due to the response-set membership of the distractor and the semantic relevance of the distractor word. In contrast, in the traditional PWI task, with many targets selected from many semantic categories, the contribution of these two components will be very small or even absent, the most important components being semantic similarity between target and distractor and lexicality. However, these differences arise from rather arbitrary choices with respect to number of semantic domains involved, the size of the response set and the response-set membership of the distractor words. They do not result from fundamental differences between the processing of colors and words. When, as we have done in the present study, the two tasks are designed to be as similar as seems possible with respect to these variables, no empirical differences between the results of the color-naming task and the picture-naming task could be observed. We conclude, therefore, that the claim that picture-word interference is a Stroop effect is warranted.^2^ In sum, the present study presents compelling evidence in favor of Glaser and Düngelhoff’s (1984) original assertion that “the color of the Stroop stimulus may be considered the limiting case of the picture component” (p. 640).

## Footnotes

1 It is interesting to note that the definition of task relevance can be extended to include other aspects of a distractor word. For example, in a PWI task in which participants are asked to name objects, distractor words of a high imageability could be argued to be more task relevant than distractors of low imageability (e.g., words with a meaning that is hard to depict, like the word *price*). Likewise, in an object-naming task noun distractors seem more task relevant than verb distractors and word distractors more task relevant than pronounceable non-word distractors. In Table 1, all of these possible components are combined in the component “lexicality.”

2 Note that this conclusion allows the construction of a picture-word test to be used in clinical practice as a replacement of the Stroop test for clients with color vision deficiency. Such deficits are quite common in men. For example, the incidence of red-green color vision deficiency is approximately 8 % among Caucasian males (Nathans, Piantanida, Eddy, Shows, & Hogness, 1986).
